# Two Pituitary Neuroendocrine Tumors (PitNETs) with Very High Proliferation and *TP53* Mutation — High-Grade PitNET or PitNEC?

**DOI:** 10.1007/s12022-021-09693-y

**Published:** 2021-10-20

**Authors:** Wolfgang Saeger, Christian Mawrin, Matthias Meinhardt, Annika K. Wefers, Frank Jacobsen

**Affiliations:** 1 Institute of Neuropathology, University of Hamburg, UKE, 20246 Hamburg, Germany; 2 Institute of Neuropathology, Otto-Von-Guericke University, 39120 Magdeburg, Germany; 3 Institute of Neuropathology, University Clinic Carl Gustav Carus, 01307 Dresden, Germany; 4 Institute of Neuropathology, University of Hamburg and Mildred Scheel Cancer Career Center HaTriCS4, UKE, 20246 Hamburg, Germany; 5 Institute of Pathology, University of Hamburg, UKE, 20246 Hamburg, Germany

**Keywords:** Pituitary, Neuroendocrine carcinoma, PitNEC, PitNET

## Abstract

We report two pituitary neuroendocrine tumors (PitNETs) with very high Ki67 labeling indices, many mitoses and *TP53* mutation (nearly all tumor cell nuclei were positive for p53). One of the tumors had bone and liver metastases. One was a corticotroph cell tumor; the other was a lactotroph tumor. The classification of these tumors is the subject of this discussion. Traditionally, pituitary carcinomas are only diagnosed by demonstration of metastases according to the 2017 WHO classification. In contrast, neuroendocrine neoplasms of the gastrointestinal tract and pancreas are classified as either well differentiated NETs that are graded as G1, G2, and G3 based on proliferation as determined by Ki67 indices of ≤ 3, 3–20 and > 20%, and/or < 2, 2–20, and > 20 mitoses per 10 high-power field respectively, or as neuroendocrine carcinomas (NECs) that are poorly differentiated neoplasms with mitoses > 20/HPF and/or a Ki67 index > 20%. With the reclassificiation of PitNETs, in our opinion, the adequate term for the well-differentiated corticotroph tumor that we report is a PitNET G3, whereas the undifferentiated prolactin tumor should be classified as PitNEC. This report expands the spectrum of pituitary neuroendocrine neoplasms.

## Introduction

The 2017 Consensus Conference of the International Agency for Research on Cancer developed the classification of neuroendocrine neoplasms (NENs) and distinguished well-differentiated neuroendocrine tumors (NETs) from poorly differentiated neuroendocrine carcinomas (NECs) [14]. This was based on the criteria used for the digestive system, which are now widely accepted (WHO classification 2019) [7]. The definitions are shown in Table 1. Whereas in NET G1 metastases are very rare [20], metastases from NET G2 are not extremely rare and metastases from NEC are frequent [5].

**Table 1:** Grading in the classification of neuroendocrine tumors (NET) and neuroendocrine carcinomas (NEC) of the digestive system [7]

**Term**	**Differentiation**	**Mitoses**	**Ki-67 index**
NET G1	Well-differentiated	< 2/10 HPF	< 3%
NET G2	Well-differentiated	2–20/10 HPF	3–20%
NET G3	Well-differentiated	> 20/10HPF	> 20%
NEC, small cell type	Poorly differentiated	> 20/10HPF	> 20%
NEC, large cell type	Poorly differentiated	> 20/10HPF	> 20%

The International Pituitary Pathology Club (IPPC) proposed the term “pituitary neuroendocrine tumors” (PitNET) [1–3] instead of pituitary adenoma. However, pituitary carcinomas remained as defined in the 2017 WHO classification by the development of metastases [12].

In our material, tumors previously classified as “pituitary adenoma” grow invasively in 49% [16] and metastasize in 0.06–0.1% of surgical specimens [15]. These properties are in contrast to the term “adenoma,” which, according to the principles of general tumor pathology, denotes a non-invasive tumor that does not metastasize. Therefore, in comparison to the gastrointestinal NET G1 and G2, PitNET is an adequate term for pituitary neuroendocrine tumors instead of adenomas [1, 2]. PitNET G1 may be used for non-invasive and non-aggressive tumors, and PitNET G2 may be used for aggressive tumors. Ki67 indices of more than 20% are very rare, and some metastasizing pituitary carcinomas show Ki67 indices of 0–16% [19] or 0 to 22% [18]; PitNET G3 as defined by a Ki-67 index of more than 20% should be very rare.

The term neuroendocrine carcinoma of the pituitary (PitNEC) is not established in pituitary pathology as poorly differentiated NECs of the pituitary (PitNECs) seem not to exist [14]. Therefore, we would like to turn your attention to two pituitary tumors with an extremely high Ki67 index and evidence of *TP53* mutation and discuss the problem of identifying PitNEC,

## Methods

Tumors were fixed in 10% buffered formalin, embedded in paraffin, and stained with hematoxylin–eosin and PAS. Immunostaining was performed with primary antibodies against GH, Prolactin, ACTH, TSH, FSH, LH, alpha-subunit, PIT1, TPIT, SF1, synaptophysin, chromogranin, S100 protein, Keratins (AE1/AE3, Cam5.2), p53, and Ki67 (MiB1).

DNA panel sequencing was performed using a self-customized targeted panel manufactured by Qiagen (CDHS-21330Z-424). This panel targets the complete coding regions and splice-sites of six genes (*ATRX*, *EGFR*, *NF1*, *NF2*, *PTEN*, *TP53*), as well as mutation hotspots of 14 further genes (*AKT*, *BRAF*, *CTNNB1*, *FGFR1*, *FGFR2*, *H3F3A*, *HIST1H3B*, *HIST1H3C*, *IDH1*, *IDH2*, *KRAS*, *PI3CA*, *PIK3R1*, *TERT*-promoter). The library was constructed according the manufacturer’s instructions. Sequencing was performed on an Illumina MiniSeq sequencing system (paired-end, 2 × 151 bp, average coverage 500 ×). Data was analyzed with the Qiagen CLC Genomics workbench, using a self-customized workflow. Variants were annotated with information from the 1000 genome project, dbSNP, ClinVar, and COSMIC. Only variants with an allele frequency ≥ 5% and a total target coverage of ≥ 40 × were analyzed further. Variants not annotated by ClinVar were additionally analyzed with VarSome (www.varsome.com).

## Results

### Case 1

A 53-year-old male suffered from Cushing’s disease with elevated cortisol and ACTH levels as well as arterial hypertension and diabetes mellitus. The pituitary macrotumor was resected transsphenoidally in an external hospital but relapsed and the patient had to undergo reoperation.

**Fig. 1:**
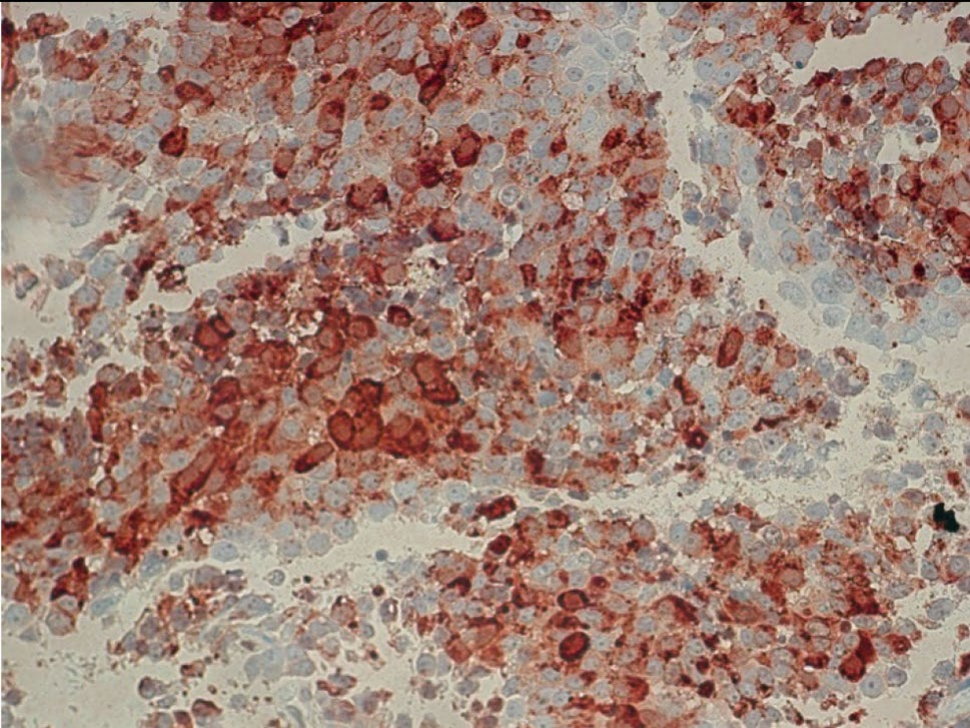
Case 1: ACTH-PitNET G3. Anti-ACTH-hematoxylin, magnification 440 ×

**Fig. 2:**
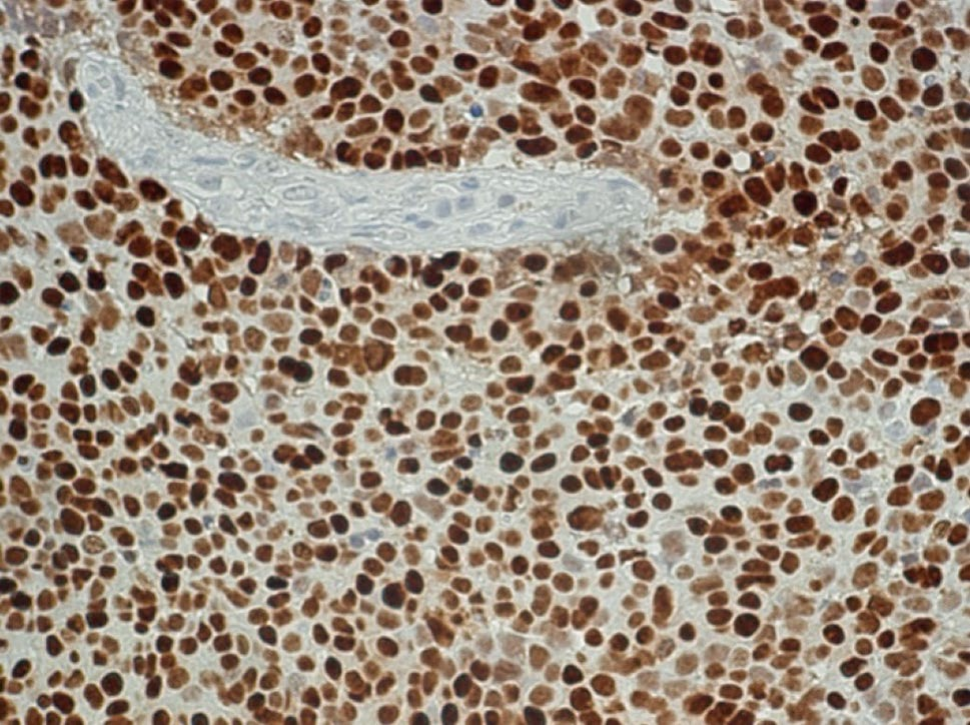
Case 1: ACTH-PitNET G3. P53-hematoxylin, magnification 440 ×

The surgical specimens showed a tumor with densely arranged medium-sized cells harboring chromatin-rich nuclei and basophilic, but PAS-negative cytoplasm (Fig. 1). Features of dedifferentiation were not evident. Mitoses were very frequent. Immunostaining for ACTH was moderately positive (Fig. 2), and the transcription factor for pituitary corticotrophs (TPIT) was expressed. The Ki67 index was very high (60–70%). P53 was localized in nearly all tumor cell nuclei (Fig. 2).

**Fig. 3:**
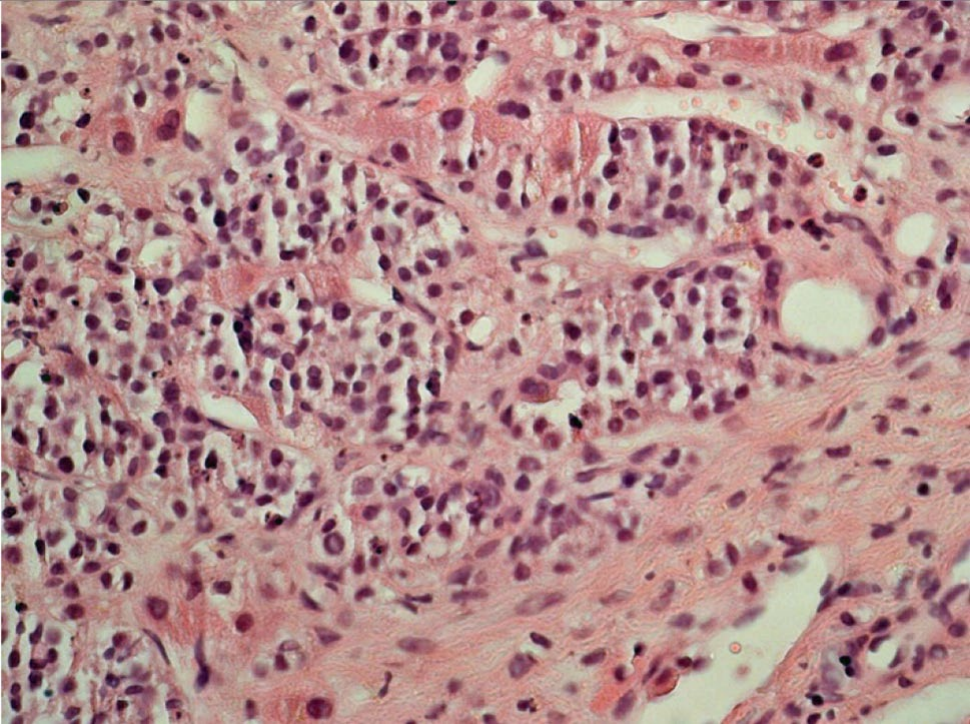
Case 1: Liver metastasis of ACTH-PitNET G3. Hematoxylin–eosin stain, magnification 250 ×

**Fig. 4:**
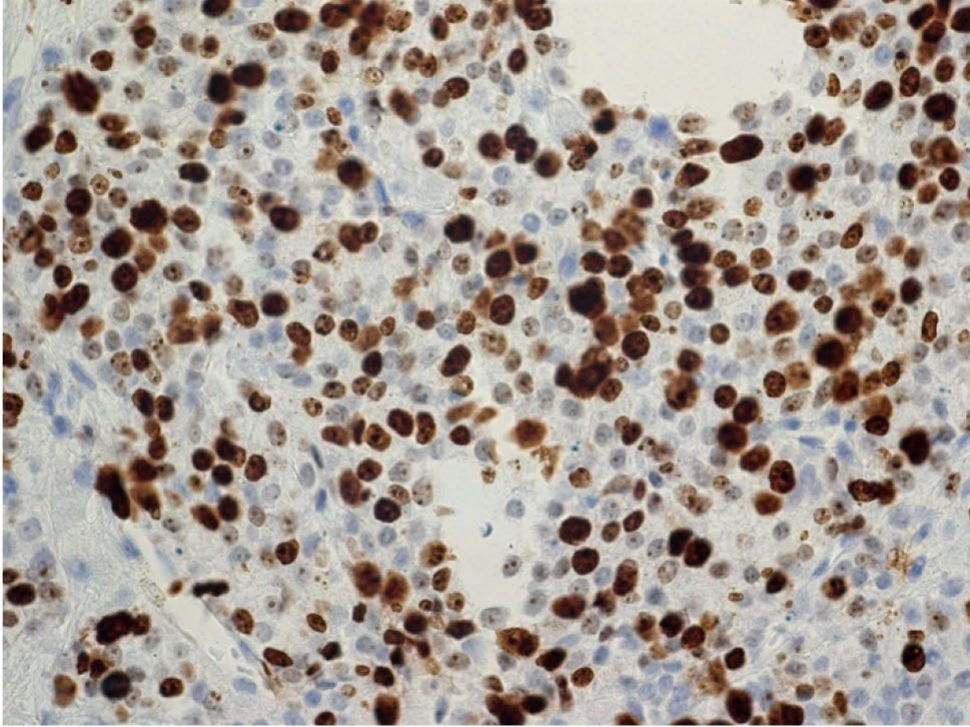
Case 1: Liver metastasis of ACTH-PitNET G3. Ki-67 (MiB-1)-hematoxylin, magnification 440 ×

After surgery, the patient received adjuvant radiation therapy (54 Gy) and systemic therapy with metyrapone (3 g) and ketoconazole (400 mg). Due to persistently increased ACTH levels, the patient was referred for a third pituitary surgery but MRI of the sellar region did not demonstrate tumor regrowth. Clinical examinations were performed to search for an ectopic source of ACTH. Thoracic and abdominal CT revealed multiple metastatic lesions in the liver and in the vertebrae. The adrenal glands were greatly enlarged. A CT-guided biopsy of the liver showed tumor tissue within and outside of the sinusoidal network as multiple, variably large foci. The histology was very similar to that of the pituitary tumor (Fig. 3). Mitoses were very frequent. The Ki67 index was just as high as in the pituitary tumor (60–70%) (Fig. 4). Immunostaining for ACTH was positive; however, in contrast to the pituitary tumor, the transcription factor TPIT was not expressed in two separate examinations. All other immunostaining was identical to the pituitary tumor (Table 2).

**Table 2:** Immunostaining of the two pituitary carcinomas

**Antibody**	**Case 1 Pituitary**	**Case 1 Liver**	**Case 2 Pituitary**
ACTH	+ + +	+ + +	0
T-Pit	+ + +	0	0
Prolactin	0	0	+ +
Pit-1	0	0	+ + +
SF-1	0	0	0
GH	0	0	0
TSH	0	0	0
FSH	0	0	0
LH	0	0	0
Estrogen receptor			+ + +
Ki-67	60%	60%	80%
P53	100%	> 95%	> 80%
Synaptophysin	+ + +	+ + +	+ + +
Chromogranin	-	-	( +)
Keratin AE1/AE3	+ + +	+ + +	-

Therefore, we can clearly state that either the tumor in the pituitary and in the liver are metastases of a tumor originating elsewhere, or the pituitary tumor is the primary tumor. This question can be answered by the expression of TPIT in the pituitary tumor, since this transcription factor is the lineage marker for pituitary corticotrophs. Moreover, we detected the same mutations of *TP53* (NM_0005465:c.743G > A) and *NF1* (NM_001042492.2:c.1318C > T) in both tumors by DNA panel sequencing. Additionally, we found two *PTEN* mutations (NM_000314.6:c.388C > T and c.210-1G > A) in the liver tumor only, as well as an *ATRX* variant of uncertain relevance (NM_000489.4:c.2044A > G).

Due to the metastases, the pituitary tumor would fulfill the 2017 WHO classification criteria for carcinoma; however, without known metastasis, this tumor would have been diagnosed as an adenoma with increased proliferation. Following the principles of NET, this relatively well-differentiated tumor should be designated as NET G3 (high grade NET).

### Case 2

A young woman aged 17 had suffered from an embryonal rhabdomyosarcoma of the orbit at the age of 3 years as well as an atypical plexus papilloma (WHO grade II) of the lateral ventricle at the age of 6 years. The rhabdomyosarcoma was treated with vincristine, actinomycin, and ifosfamide. The plexus papilloma was treated by gross total resection.

Eleven years later, a tumor in the sellar region was diagnosed and transsphenoidally resected with a presumptive diagnosis of recurrent rhabdomyosarcoma. Preoperative prolactin levels were moderately increased (762 mU/L, reference range: 102–496). One week after surgery, the prolactin level was 653 mU/L, and 6 weeks after surgery, it remained at 653 mU/L.

**Fig. 5:**
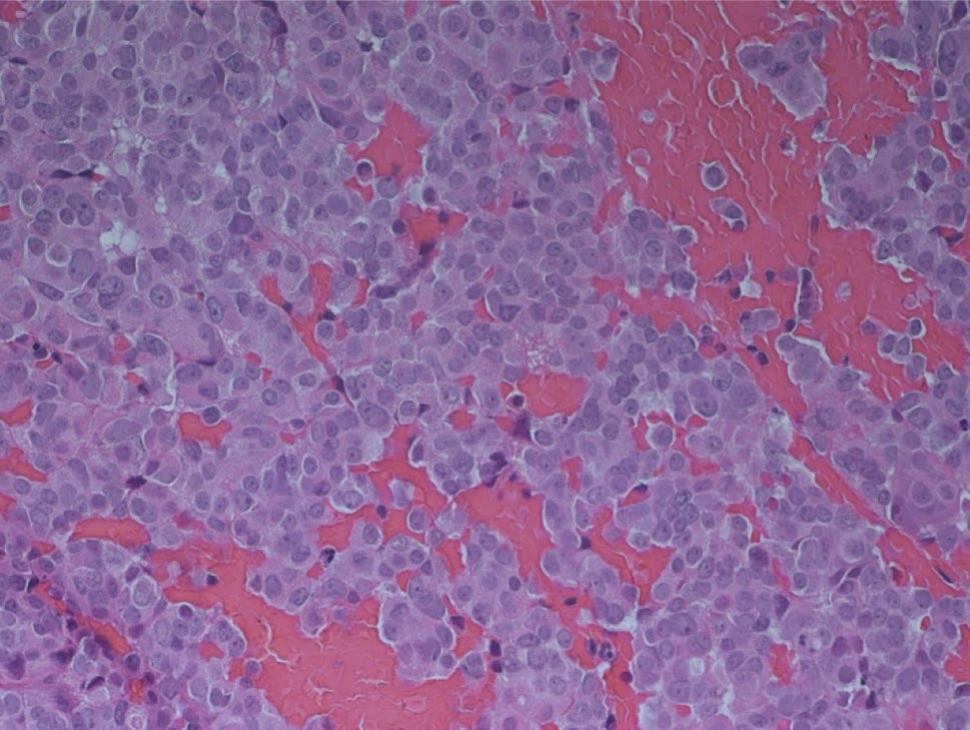
Case 2: Prolactin-PitNEC. Hematoxylin–eosin stain: magnification 440 ×

**Fig. 6:**
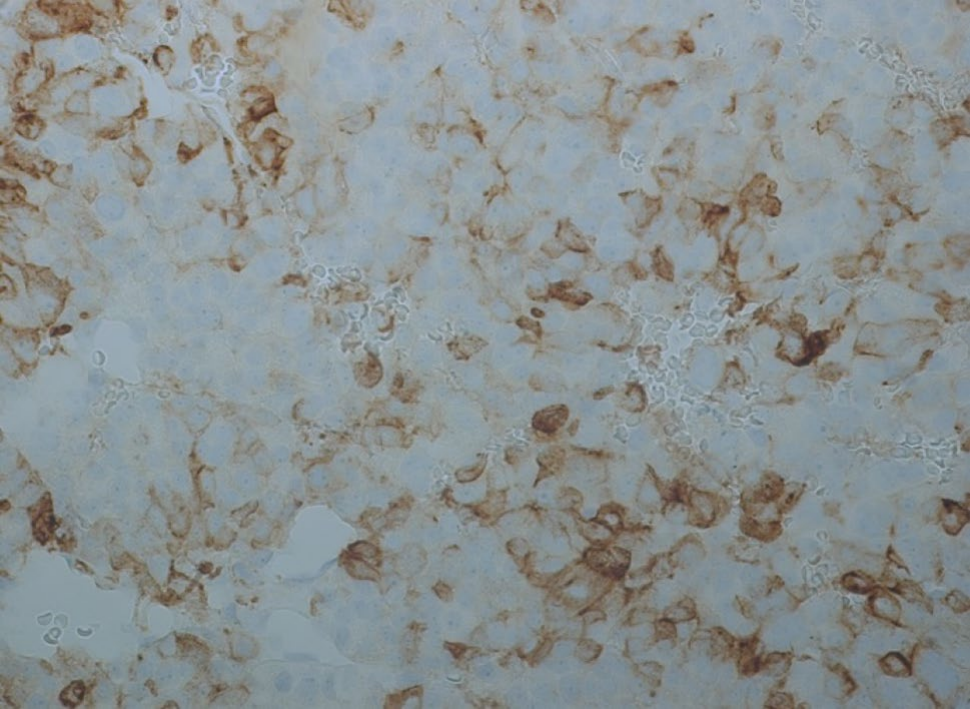
Case 2: Prolactin-PitNEC. Anti-Prolactin-hematoxylin, magnification 440 ×

**Fig. 7:**
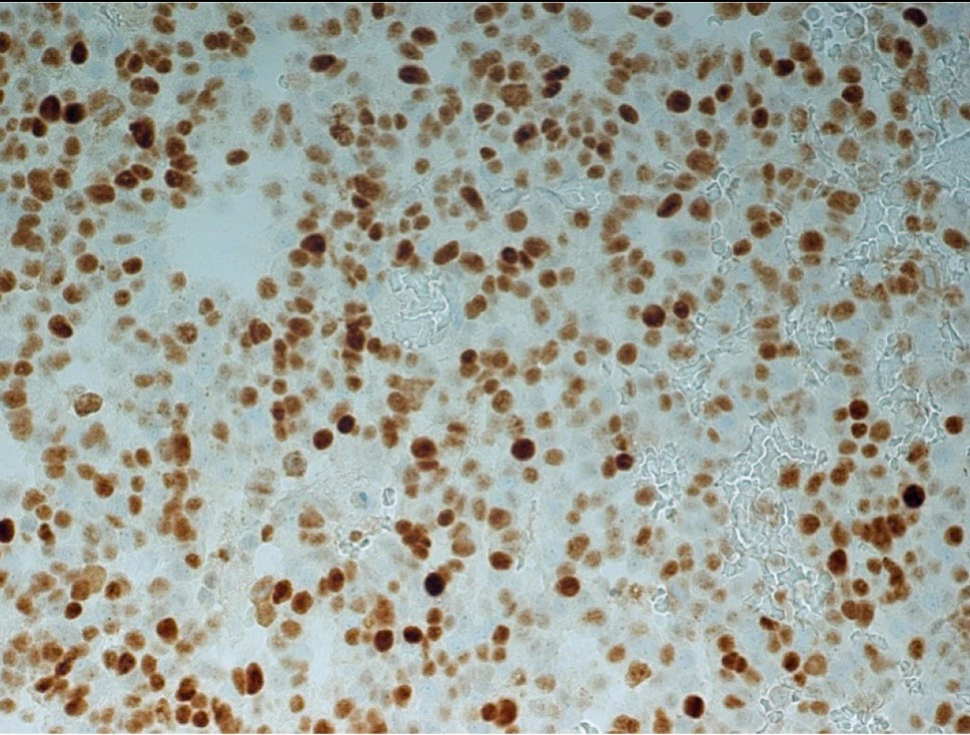
Case 2: Prolactin-PitNEC. P53-hematoxylin: magnification 440 ×

**Fig. 8:**
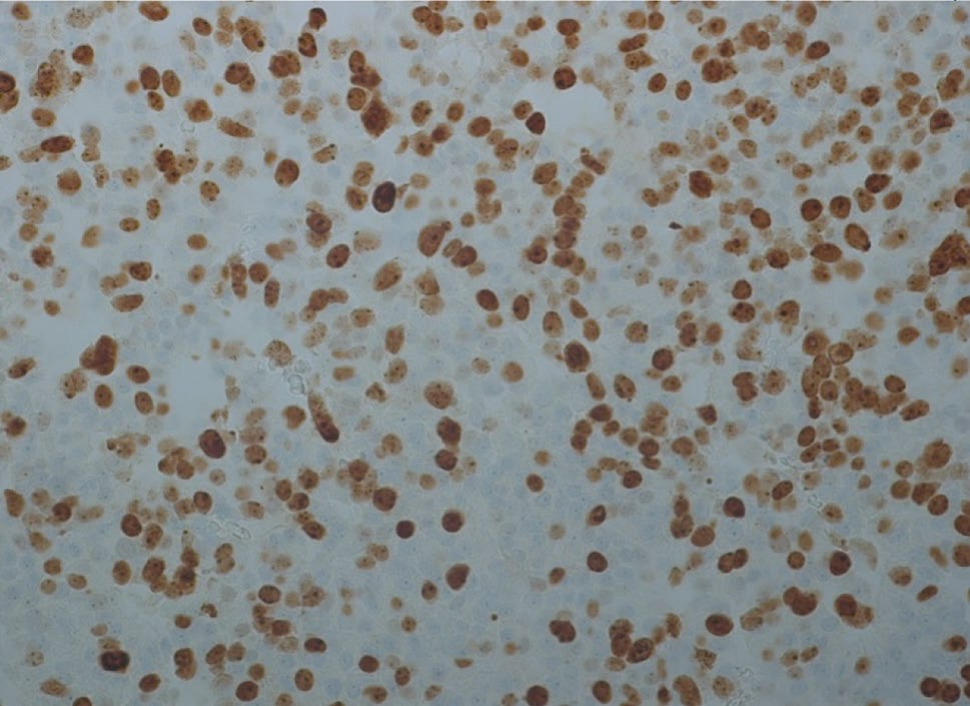
Case 2: Prolactin-PitNEC. Ki-67 (MiB-1)-hematoxylin: magnification 440 ×

The sellar lesion was a pituitary neuroendocrine tumor with diffuse arrangement of small to medium-sized cells and many mitoses (Fig. 5). Immunostaining (Table 2) revealed strong expression of PIT1, moderate expression of prolactin (Fig. 6), and strong nuclear expression of estrogen receptor. Nuclear staining for p53 was intense (Fig. 7), and the Ki67 index exceeded 50% (Fig. 8). DNA panel sequencing revealed a *TP53* mutation (NM_000546.5:c.731G > A).

Following the principles of NET, in contrast to a highly differentiated sparsely or densely granulated lactotroph tumor, this neoplasm was composed of smaller cells without the typical immunostaining pattern for prolactin (Golgi pattern in the sparsely granulated type or strong diffuse staining in the densely granulated type). Due to the lack of these hallmarks, this tumor should be designated as PitNEC or at least as a questionable PitNEC.

## Discussion

One problem regarding the histopathology of case 1 was the negative staining for TPIT in the liver metastasis, whereas ACTH was positive. In the pituitary primary tumor, TPIT and ACTH were strongly expressed. We have no explanation for this fact, since an ACTH-expressing pituitary tumor must express TPIT [17]. It may be that the harboring tissue (liver) plays a role.

Two cases published in the literature can be compared with our tumors: (1) The case report of Pasquel et al. (2013) [13] described a nonfunctioning PitNET with a Ki67 index of 30 to 80%. Due to the lack of metastases, the authors proposed the term “carcinoma in situ” for this tumor. (2) Guo et al. [6] found an ACTH-expressing metastatic pituitary neoplasm with *TP53* mutation and novel gene mutations in *ATRX* and *PTEN* genes. The tumor showed high pleomorphism, numerous mitoses, very strong nuclear expression of p53, and a Ki67 index of up to 80%. This tumor appears very similar to our case 1.

Due to the high Ki67 index, the high number of mitoses, and the extremely strong expression of p53 in the nuclei, both tumors in this study differ from pituitary carcinomas described in the literature and in the 2017 WHO classification [12]. According to the gastrointestinal NETs, both tumors can be named PitNET G3 if they are well differentiated, or PitNEC when they are poorly differentiated. The ACTH-producing neoplasm (case 1) appeared to be well differentiated due to the relatively large cytoplasm, but the prolactin-producing neoplasm (case 2) had scanter cytoplasm and lacked other signs of lactotroph differentiation; therefore, it appeared to be a poorly differentiated neoplasm. These facts show that both NET types — the PitNET G3 and the PitNEC — may exist if the NET principles are applied in pituitary tumor pathology. Casar-Borota et al. [4] examined 30 aggressive PitNET and 18 pituitary carcinomas and found negative immunostainings for *ATRX* protein (Alpha thalassemia/mental retardation syndrome, x-linked) in 13% of aggressive PitNETs and in 28% of carcinomas. Additionally, they found a strongly increased risk for clinically silent corticotroph PitNETs to have metastatic dissemination.

The demonstration of p53 in pituitary tumor nuclei was described by Kovacs et al. (2013) [8], but this tumor showed 15 Ki67 positive nuclei in 10 high power fields, which does not correspond to a very high Ki67 index. Further studies describe the Ki67 index but not the P53 status: MacCormack et al. (2018) [11] found Ki67 indexes between 10 and 38% in 40 pituitary carcinomas. Thapar et al. (1996) detected K67 indexes between 0 and 22% in 7 carcinomas [18], or Ki67 indexes between 0 and 16% [19]. A case report from Lin et al. [9] deals with a pituitary ACTH-producing PitNET causing Cushing’s disease with metastasis to the liver that manifested a mitotic index of up to 50%. Molecular pathology revealed an amplification of *CCND3* (Cyclin D3 protein coding gene), homozygous deletion of *PTPRD* (protein tyrosine phosphatase, receptor type D), and in the liver pathway, activation of PI3K (Phosphoinositid-3-Kinasen) via subclonal *PIK3CA* G1050D hot spot mutation. Next-generation sequencing by Majd et al. [10] revealed gene alterations in four pituitary ACTH-producing carcinomas after therapy with pembrolizumab.

Pituitary pathologists should discuss the distinction of NET G3 and NEC. We propose to delineate all differentiated pituitary tumors with a Ki67 index of more than 50% and the presence of p53 mutation as PitNET G3, as most of these develop metastases, and the undifferentiated/poorly differentiated tumors as PitNEC. Then clinicians can formulate guidelines for postoperative control examinations and therapies. The old principle of referring to tumors as carcinomas according to the existence of metastasis appears absolutely unsuitable for patients and clinicians.
